# Antioxidant Potential of Glutathione and Crosstalk with Phytohormones in Enhancing Abiotic Stress Tolerance in Crop Plants

**DOI:** 10.3390/plants12051133

**Published:** 2023-03-02

**Authors:** Gyanendra Kumar Rai, Pradeep Kumar, Sadiya M. Choudhary, Hira Singh, Komal Adab, Rafia Kosser, Isha Magotra, Ranjeet Ranjan Kumar, Monika Singh, Rajni Sharma, Giandomenico Corrado, Youssef Rouphael

**Affiliations:** 1 School of Biotechnology, Sher-e-Kashmir University of Agricultural Sciences and Technology of Jammu, Jammu 180009, India; 2 Division of Integrated Farming System, ICAR—Central Arid Zone Research Institute, Jodhpur 342003, India; 3 Department of Vegetable Science, Punjab Agricultural University, Ludhiana 141004, India; 4 Department of Biotechnology, BGSB University, Rajouri 185131, India; 5 Division of Biochemistry, ICAR—Indian Agricultural Research Institute, New Delhi 110001, India; 6 GLBajaj Institute of Technology and Management, Greater Noida 201306, India; 7 Department of Agronomy, Punjab Agricultural University, Ludhiana 141004, India; 8 Department of Agricultural Sciences, University of Naples Federico II, 80055 Portici, Italy

**Keywords:** antioxidants, abiotic stress, reactive oxygen species, hormone crosstalk, Jasmonic acid

## Abstract

Glutathione (GSH) is an abundant tripeptide that can enhance plant tolerance to biotic and abiotic stress. Its main role is to counter free radicals and detoxify reactive oxygen species (ROS) generated in cells under unfavorable conditions. Moreover, along with other second messengers (such as ROS, calcium, nitric oxide, cyclic nucleotides, etc.), GSH also acts as a cellular signal involved in stress signal pathways in plants, directly or along with the glutaredoxin and thioredoxin systems. While associated biochemical activities and roles in cellular stress response have been widely presented, the relationship between phytohormones and GSH has received comparatively less attention. This review, after presenting glutathione as part of plants’ feedback to main abiotic stress factors, focuses on the interaction between GSH and phytohormones, and their roles in the modulation of the acclimatation and tolerance to abiotic stress in crops plants.

## 1. Introduction

The tripeptide thiol molecule glutathione (GSH) with an amino acid composition including cysteine, glutamic acid, and glycine is a common and widespread antioxidant in plants and plays an important role in stress response as a scavenger of free radicals. Reactive oxygen species (ROS) are a group of highly reactive molecules that are considered an unavoidable metabolic product of aerobic organisms, present in low and relatively stable amounts under normal physiological conditions. Oxidative stress occurs because of increased ROS accumulation or due to higher production and/or insufficient detoxification. Plants have evolved specific pathways and molecules to protect cells from ROS toxicity. GSH is one of the most critical components, being ubiquitous, present in different subcellular compartments, relatively abundant, and involved in a variety of cellular events, from the synthesis of DNA and proteins to cellular defense [1]. GSH is found in two forms: (1) the reduced form (GSH), considered one of the most important ROS scavengers, and (2) the oxidized form (GSSG), which then reverts to reduced glutathione by the enzyme glutathione reductase. There is a prevalence of GSH over GSSG under normal conditions. Cellular toxicity can be determined by the ratio of reduced versus oxidized glutathione [2].

**Figure 1:**
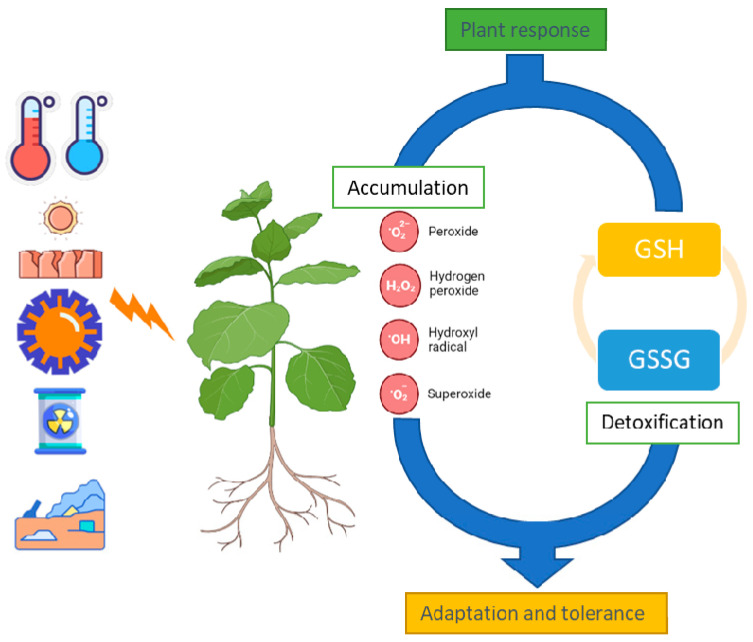
A simplified view of the role of GSH in the redox homeostasis in plants following abiotic stress (such as extreme temperatures, drought, solar radiation, toxic elements, and poor soil conditions) and the subsequent cellular increase in common oxidants.

Abiotic stress deriving from extreme temperatures, nutrient availability, water quality and accessibility, soil characteristics, unsuitable radiation, and toxic elements causes major crop loss [3]. Symptoms may be different and include withering, chlorosis, reduced growth, altered development, wilting, organ (e.g., leaf, flower, fruit) abscission, and rot/necrosis [4]. During stress conditions, ROS act in plants as transduction molecules that control different pathways. In addition to being biochemical products of the (stress) metabolism, NADPH oxidases (also named respiratory burst oxidase homologs, RBOHs) are a major source of ROS in stressed plants along with other oxidases and peroxidases [5]. The primary adaptive response to oxidative plant stress is an increase in antioxidant defense system activity [6]. This includes the participation of various antioxidants which are non-enzymatic such as GSH, ascorbic acid (AsA), tocopherols, phenols, other secondary metabolites, and inorganic amino acids, which have the common role of maintaining redox homeostasis [7]. In addition, several antioxidant enzymes, such as monodehydroascorbate reductase (MDHAR), glutathione reductase (GR), dehydroascorbate reductase (DHAR), glutathione S-transferase (GST), and glutathione peroxidase (GPX) are involved in these defense responses [8]. For instance, GSTs promote the conjugation of GST with xenobiotics, superoxide dismutases partition the superoxide radical in oxygen and hydrogen peroxide, catalases decompose hydrogen peroxide into water and oxygen, peroxidases (POD) catalyze the oxido-reduction between hydrogen peroxide and reductants, and MDHAR, DHAR, ascorbate peroxidase (APX), and GRs are members of the ascorbate–glutathione cycle that detoxifies hydrogen peroxidase [9] (Figure 1). GSH additionally reduces dehydroascorbate DHA into AsA in the form of antioxidants in the AsA–GSH cycle, and in different plant species, it has effect of cold resistance [10].

Phytohormones are molecules present in small amounts that can influence physiological processes in plants [11]. Phytohormones are chemical messengers that help in the management of cellular functions and signaling [12]. Auxins were the first discovered phytohormones [13] while strigolactones (SL) are the most recently identified [14]. Although classifications can be slightly different, the commonly recognized phytohormones are abscisic acid (ABA), brassinosteroids (BR), gibberellins (GA), jasmonates (JA), cytokinins (CK), auxins, salicylates (SA), strigolactones (SL), and ethylene (ETH) [12]. Some phytohormones mediate plant defense response to abiotic stress such as JA, ET, ABA, and SA [15]. Environmental conditions including wounding, cold, heat, salinity, and drought stresses mostly cause increases in abscisic acid levels; this is because abscisic acid is usually accountable for plant defense towards abiotic stresses [16]. Regarding regulating plant defense response, a recent study provides considerable proof for the interaction of JA, SA, ET, and ABA with CKs, Gas, and auxins [17]. At the transcriptional level, the phytohormonal impact on GSH is not well-described, while it is well-known that GSH action requires some phytohormones. In Arabidopsis (*Arabidopsis thaliana*), the exogenous supply of GSH improved tolerance to salt and heavy metal (lead) stress in wild-type Col-0 and not in the ET-signaling mutant *ethylene*-*insensitive protein 2-1* (EIN2) gene [18].

## 2. GSH Biosynthesis

**Figure 2:**
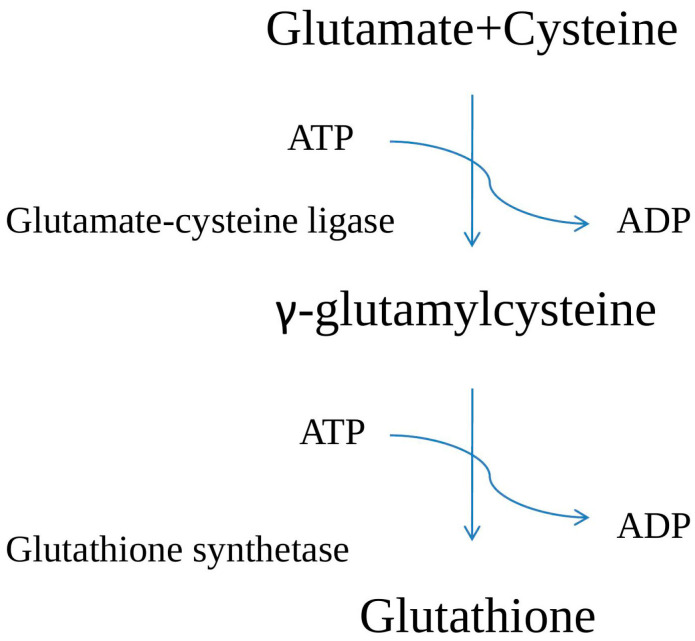
Glutathione biosynthesis in plants.

The biosynthesis of GSH encompasses two ATP-dependent stages (Figure 2). In the first, glutamate–cysteine ligase (GCL) catalyzes the reaction between glutamate and cysteine to produce γ-glutamylcysteine. By the addition of glycine to γ-glutamylcysteine, glutathione is produced via glutathione synthetase. The balance between GSH and GSSG is critical for preserving the cell’s homeostasis [2,19]. The availability of GCL and GS plays a crucial part in glutathione biosynthesis. GCL and GS are localized in the chloroplast and cytosol, respectively [20]. In Arabidopsis, glutamate–cysteine ligase and glutathione synthetase (GS) are encoded by the same gene (with different start sites), which produces proteins that are localized into the cytosol or transported to plastids [21]. Plant systems are unique in that they compartmentalize glutathione biosynthesis [2,22]. In plants, glutathione content is typically increased by over-expression of GCL rather than GS by increasing flux through the system. Arabidopsis seedlings treated with 5 mM H_2_O_2_ demonstrated an elevation in GCL function that ranged from GSH to GSSG form, as shown by immunoblot and activation experiments [23]. Furthermore, the importance of GCL as a metabolic control point is supported by the fact that the accumulation of glutamate, cysteine, or glycine does not significantly enhance glutathione synthesis when in non-limiting/stressed conditions [24,25]. The glutathione production pathway is tightly regulated, and it is generally accepted that GCL and GS genes are upregulated under a variety of stresses [26].

**Figure 3:**
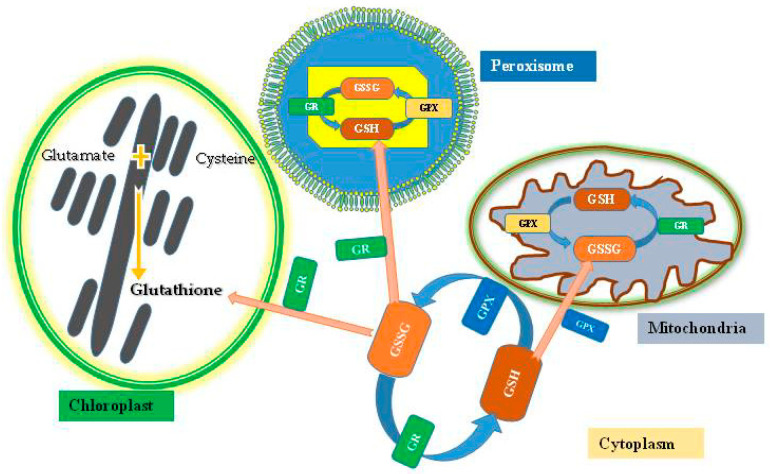
Diagrammatic illustration of biosynthesis pathways of glutathione in chloroplast, peroxysome, and mitochondria.

Glutathione can also be described as a non-protein-reduced sulfur. GSH synthesis occurs in the cytosol, as well as in mitochondria, peroxisome, and chloroplasts (Figure 3) [27]. There are three amino acids initiating the reaction: glycine, cysteine, and glutamate. Two proteins catalyze the GSH synthesis (glutathione synthase (GSHS) and c-glutamylcysteine synthetase (cECS)). Plastids and cytosol are found in GSH while cECS is only found in plastids; because of this, in higher plants, the location of GSH creation is plastids [28]. Initial GSH biosynthesis comprises a response flanked by an “a-amino group” of the amino acid cysteine and a “c-carboxyl group” of the amino acid glutamate and constructs an amide bond by providing cECS, which ultimately brings about this reaction. The subsequent advance is trailed by the GSH arrangement, and it happens within the sight of catalyst GSHS by the development of amide connection among the a-amino part of glycine with the c-carboxyl part of cysteine amino acid in c-glutamylcysteine, which is consequently GSH-framed [20,29]. 

Under stress conditions, the transformation of reduced GSH into GSSG can happen inside various compartments through biochemical responses. The GSH-to-GSSG ratio is dependent mainly on the activity of glutathione reductase and also on glutathione peroxidases (GPX) [30]. Glutamate donates the carboxyl part and cysteine donates the amino part and, thus, the peptide bond connecting them is created. Subsequently, γ-glutamylcysteine accumulates glycine to contribute in glutathione synthesis. There is also the presence of a peptide bond between them. Glutathione synthetases (GS, GSH-S, GSH2) catalyze the formation of GSH. However, this reaction is dependent on ATP. The location of the GSH2 catalyst is mainly in plastids as well as in the cytosol, whereas the GSH1 enzyme is found in plastids and significantly controls GSH biosynthesis [5].

## 3. GSH as Regulatory and Antioxidant Molecule

It is well-known that GSH is an important antioxidant molecule [31]. In plants, the presence of GSH-specific peroxidases has been problematic because they, as indicated by GPXs in plants, show no reaction with GSH—only by means of TRXs (thioredoxins) [32]. GSH detoxifies lipid peroxides, methyl glyoxal, and pesticides in addition to H_2_O_2_ [33,34]. First, one ATP molecule is used by the GCL during the formation of glutamylcysteine from glutamate and cysteine. After that, glycine is transferred to the dipeptide catalyzed by GS and also requires another ATP molecule. It is interesting to note that oxidative stress (OS) can activate the nuclear factor erythroid 2-related factor 2, which controls GCL synthesis. As a result, oxidative stress increases GSH synthesis by promoting GCL action [35]. Glutamyl bonding, which makes GSH exceptionally robust and resilient to degradation by most proteases and peptidases, is one of the distinguishing physical features of GSH. The enzyme gamma-glutamyl transferase (GGT) breaks down the extracellularly situated GSH by removing gamma-glutamyl and, hence, produces cysteinylglycine or cysteinylglycine conjugates, which are further broken by dipeptidases [36].

Stress-instigated variations in GSH content as well as GSH/GSSG proportions could be due to a shift in GSH synthesis. The formation of cEC is mediated by cECS first and, subsequently, GSH synthetase adds a glycine to the dipeptide. The cECS is an administrative catalyst of glutathione amalgamation [37]. In addition to GSH, hGSH (homoglutathione) has been found in of the Fabaceae family [38], and in the Gramineae family, the presence of hmGSH (hydroxymethyl glutathione) is seen, but the biosynthetic pathway has not yet been determined [39]. The absorption of 35S via solute onto GSH as well as the augmented movement of the two proteins linked with GSH production showed that the cold-actuated expansion of maize in total glutathione (TG) concentrations is the direct consequence of a faster synthesis rate [40]. Furthermore, freezing stimulated cECS action and cEC concentration in maize leaf bundle sheath cells [41]. Elevated temperature, cold treatment, and osmotic pressure initiated more noteworthy expansion within GSH in addition to hmGSH production in resistant hybrids of wheat as compared to the sensitive ones, as displayed in 35S-marked experiments [42].

Since there have been comparative contrasts in the ratios of GSH/GSSG and hmGSH/hmGSSG or, rather, their reduced/oxidized antecedents among grain yields of tolerant and sensitive wheat exposed to heat stress or osmotic stress, abiotic stress-initiated changes (as in redox conditions of GSH substrates) might impact the amalgamation and redox status of GSH [42,43]. In peas, induction of glutathione reductase activities via heat stress was observed and was not due to GR gene expression [44]. The amassing of c-glutamylcystine, the oxidized type of the GSH antecedent in tobacco overexpressing cECs (c-glutamylcystine), demonstrates that no existing chemical has been found for its effective reduction in plants; thus, it can be detached exclusively by sequestration/degradation to the vacuole [45].

The linkage among antioxidants and ROS might provide metabolic contact points. These points are found in-between signals from metabolic pathways as well as those from the climate, directing the acceptance of processes of apoptosis [31]. The antioxidant activity framework, which includes the GSH/GSSG redox reaction, could have evolved to modulate redox signaling and the cellular redox state, as well as to organize gene expression [46]. During abiotic stress, this network regulates the amount of ROS produced by combining signals from diverse cell components, and the GSH/GSSG ratio aids in its tweaking [47]. Research reports demonstrated the role of natural redox and signaling chemicals in reducing abiotic stressors, such as harmful metals in plants. These compounds include reduced GSH and hydrogen sulfide (H_2_S) [48,49].

## 4. Role of GSH under Abiotic Stress in Plant System

Due to the anthropogenic changes in the environment such as climate change, abiotic stress has become an increased threat to food security. Plants can react and adjust to abiotic stress by undergoing a variety of physiological, molecular, and cellular modifications. [50].

### 4.1. Oxidative Stress

GSH is the main antioxidant that directs abiotic stress reactions [51,52]. In cells, it additionally balances out redox homeostasis, invigorates stress-associated signals, promotes stress endurance, and detoxifies xenobiotics [53]. GSH initiates glutathionylation so that it can use oxidative stress for protein shielding [54,55]. In plants, this information could be utilized for the indication of stress markers to recognize necrotic cues [56]. Because of the abiotic stress, the GSH concentration additionally fluctuates inside various subcellular segments. Organelle-explicit varieties are shown by this tripeptide thiol during stress because of its assorted jobs within distinctive cell components. GSH maintains the declining interior climate inside cells and, furthermore, restricts uncontrolled oxidation of proteins and membranes [57].

Hence, whether stress is prompted impermanently and slightly deviates from the typical shape, or causes more extreme and lasting harm, is mediated by the defensive system by GSH aggregation which is, for the most part, found in plants during stress [58]. Reduction in H_2_O_2_ is a primary capacity of GSH against oxidative stress security [5,31,59]. During certain redox signaling pathways, this reduction has extraordinary significance regarding the transformation of GSH into GSSG at the time of H_2_O_2_ degradation [5,60].

Due to the extreme increase in the levels of ROS, a large grouping of GSH is utilized for scavenging them; thus, GSSG ultimately has the higher proportion [61]. A high GSH/GSSG ratio is interceded by GSH reductase. For keeping up protein construction and function, lessening the intracellular climate is significant. In the formative stage, the GSH/GSSG proportion shifts. For working with somatic embryogenesis, the GSSG/GSH proportion prerequisite is high [62]. Here, molar concentrations in relation to GSSG and GSH that comprise GSH half-cell reduction potential, at the time of stress, act as a basic indicator of cell feasibility. During formative and stress reactions, the assurance of redox balance in chloroplasts is finished by the plastoquinone pool alongside changes in the GSH redox state as well as AsA. The chloroplast retrograde pathway recognizes the redox signaling as well as ROS, which are intervened by H_2_O_2_. In plasma membrane GSH, the electron donor is needed for AsA-subordinate redox transport. Moreover, redox homeostasis is ultimately dictated by the calibration of the GSH/GSSG proportion, and it helps plants during imperfect conditions for endurance [63].

**Figure 4:**
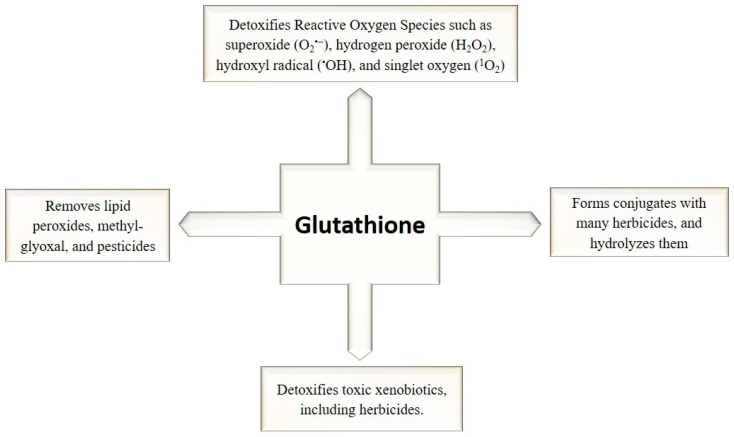
Glutathione‘s role as a master antioxidant in plant defense system.

During abiotic stress, vacuoles act as sinks for ROS. The rate-limiting enzyme GR which mediates the AsA–GSH cycle ensures elevated AsA concentrations in the vacuoles to easily scavenge phenoxy radicals [64]. The cat2 mutant competes with excessive GSH aggregation in the vacuoles to avoid the negative consequences of GSSG gathering. These include arrangement of necrosis, torpidity, and sores [65]. The overabundance of ROS in the vacuoles is detoxified by GSH during abiotic stress and for extraction of huge measure of GSH from cytoplasm goes about as a sink [66]. Glutathione combats various abiotic stresses by using its antioxidant defense machinery. Usually, it acts as a master antioxidant, as depicted in Figure 4. 

### 4.2. Heat Stress

Heat stress negatively impacts plants’ performance. GSH application, however, improves plants’ ability to cope up with heat stress by modulating physiological and metabolic functioning, chiefly through the induction of antioxidant enzymes in combination with ROS scavenging. External GSH administration helped preserve leaf moisture content with enhanced antioxidant enzyme activity, thereby inducing resistance to heat stress in plants [67]. 

Few researchers have looked into the positive effects of GSH on plants’ performances under heat stress (Table 1). Important elements of thermotolerance include GSH pools, with their seasonal and geographical variations, as well as the GSH redox state, with its control and functions in redox signaling and defense activities [68]. The response of maize to heat stress at the seedling stage is linked to the GSH pool. Heat stress (40 °C, 1 day) pre-treatment raised total GSH levels and the GSH/GSSG ratio in chilling-sensitive inbred lines, although total hydroxyl methylglutathione concentration and GSH reductase activity was enhanced in both tolerant and chilling-sensitive maize genotypes [69]. Plants of *Coleus blumei* and *Fagus sylvatica* were subjected to oxidative stress brought on by heat stress (35 °C). The seedlings of both species, even if in diverse ways, displayed variations in the GSH pool as well as in GR activities after a brief period of acclimatization to low temperatures, which was connected with the thermal stress tolerance among these species. *F. sylvatica* also showed greater heat tolerance since *Coleus blumei* had lower GSH levels and GR activity. It was discovered that *C. blumei’s* heat susceptibility was linked to a differential depletion of GR and GSH at cold temperatures [70].

It was hypothesized that wheat, maize, and green gram would be more tolerant to heat if their total GSH concentration was higher [71,72]. The increased GSH level in mustard seedlings under high temperatures was achieved by higher GR activity, which allowed for the removal efficiency of H_2_O_2_ [73]. Furthermore, in apple plants at the reproductive stage, higher GSH levels improved their ability to acclimatize to heat stress. Increased endogenous GSH levels were found in apple peels that had been exposed to heat and intense sun radiation, which enhanced their ability to adapt to heat stress. When ascorbic acid, benzoic acid, and salicylic acid were applied exogenously, endogenous glutathione content was raised by 33.97 and 31.81%, respectively, in contrast to the control, while acclimatization raised it further [74]. *Malus domestica* Borkh plants that were two years old when subjected to a temperature of 40 °C for 0, 2, 4, 6, and 8 h showed an increase in the AsA–GSH process after exposure to heat. Under heat exposure, after two hours, the highest levels of total GSH, as well as total and decreased AsA concentrations, were noted. For up to 4 h of heat stress, ascorbate peroxidase (APX), DHAR, and GR activity and transcription were enhanced. As a result, *M. domestica’s* overexpression of the AsA–GSH cycle demonstrated preventive properties against heat stress-induced oxidative damage throughout the vegetative growth stage [75]. In *Soldanella alpina* as well as in *Ranunculus glacialis*, higher total GSH concentration enhanced antioxidant defense and electron utilization, which in turn improved heat stress resistance [76].

### 4.3. Cold Stress

Cold stress causes foliar chlorosis and a decline in the quality of the function and structure of cells and tissues by delaying leaf development, prolonging the cell cycle with reduced cell production, and stunting growth. To fight cold stress, external GSH administration was found to be beneficial via reducing lipid peroxidation and electrolyte leakage [77]. In comparison to untreated cold-stressed plants, rice plants sprayed with 0.5 mM GSH had longer shoots and roots, higher fresh weight, as well as higher levels of endogenous GSH (Table 1).

Tomato is an important vegetable crop due to its beneficial composition, which possesses antioxidant properties [78]. However, tomatoes, similar to many other crops, are chilling-sensitive, which places them in the category of cold-susceptible crop varieties [79]. Depending on the genotypic background, the normal temperature for fruit set ranges from 15 to 25 °C. Reduced fruit set at low temperatures is caused by poor anther dehiscence, pollination, and pollen viability, among other factors. Many commercial cultivars are vulnerable to low temperatures across all developmental stages, from seed germination through to vegetative growth and reproduction [80].

Low temperature changes the physiology of plants, causing damage to their cell membranes, changes in their lipid composition, chlorosis, and different enzyme activities that cause necrosis and even death in some cases [81,82]. The level of tolerance of the Pusa Sheetal cv. of tomato is decreased by cold stress. Exogenous GSH supplementation, however, has the tendency to raise the level of the tolerance [83]. During cold acclimation, an increase in total glutathione content can be observed as an after-effect of diminished degradation. Modifications in GSH metabolism potentially alter TG content in stressful situations, but stress also causes changes in the transfer of GSSG or GSH from one organ to the next [84]. The formation and migration of GS formed during the evacuation of something, such as the hazardous consequences of lipid peroxidation occurring at low temperatures, might also affect GSH concentration [84]. Collectively, these outcomes provide insight into how increased GSH levels due to decreased degradation or expanded synthesis may improve resistance against stress at low temperature.

### 4.4. Salinity Stress

Salinity stress gives rise to a decrease in water uptake, photosynthesis, and seed germination, nutritional imbalances, salt ion toxicity, and a general decline in agricultural output. Salinity stress can be treated with GSH administration. This improves plant growth, the fresh and dry masses of shoots and roots, and total yield. To counteract salinity stress, exogenous administration of GSH has boosted water usage efficiency (WUE), antioxidant enzyme activity, and levels of osmoprotectants [85]. Foliar sprays with 0.4 and 0.8 mM of GSH enhanced WUE, dry and fresh weights of shoots and roots, and the concentrations of osmolytes and antioxidants in pepper plants under salinity stress (Table 1).

Plant species such as rice [86], tobacco [87], tomato [88], Arabidopsis [89], *Brassica chinensis* [90], and *Nicotiana* exhibited the common salt stress response known as redox imbalance (GSH/GSSG and AsA/DHA) [91]. Although it has been extensively documented how stress-induced redox reactions contribute to plant oxidative damage, it is also well-known how plant redox reactions are regulated [92,93]. It was concluded that GSH is linked to the control of redox during cell cycles and improves adaptation to a variety of abiotic stresses, especially salt stress [94,95]. Numerous species have been subjected to extensive research regarding the GSH production pathway in vegetable crops, which is made up of numerous enzymatic stages [96,97]. GSH is transformed into GSSG as an important component of cellular antioxidant defense. The lowered GSH/GSSG state is then maintained by GSH reductase (GR) in conjunction with NADPH. When salt stress triggers ROS detoxification, the redox state (GSH/GSSG) and the expression of the genes encoding glutathione S-transferase (GST), GPX, and GR play crucial roles [98,99].

Studies evaluating the susceptibility of wound-induced protein (WIP) family members to salt in plants are not yet available. WIPs may, however, be connected to tomatoes’ GSH and salt-mediated antioxidant capabilities. Research investigations demonstrated the thorough identification of WIPs and investigated a novel mechanism for zinc-finger protein (ZFP)-type WIPs in controlling salinity tolerance [100]. 

### 4.5. Heavy Metal Stress

Heavy metal stress includes various heavy metals such as cadmium, nickel, lead, mercury, copper, zinc, etc. In general, heavy metal stress has harmful effects on plants, including reductions in growth, photosynthesis, and nutrient uptake, as well as changes to the water balance, chlorosis, and senescence. On the other hand, it has been found that applying glutathione to plants increases their ability to withstand stress by boosting their antioxidants, ROS scavenging, and photosynthetic pigment levels. Glutathione was applied exogenously to increase photosynthetic pigments and reduce oxidative damage in order to counteract the negative consequences of heavy metal stress [101]. In wheat, plants exposed to cadmium (Cd) stress as a result of heavy metal toxicity have higher levels of endogenous GSH and photosynthetic pigments (Table 1).

Under Cd stress, plants increased the amount of GSH quickly and chelated Cd^2+^, thereby reducing the harm that Cd stress caused to the cytosol’s metabolic processes [102]. Under stress conditions, phytochelatin synthase (PCs) may transfer c-Glu-Cys to GSH, and subsequently form phytochelatin (PC) [103]. PC can, indeed, integrate Cd^2+^ directly, and can also lessen the harm of oxidative damage induced from heavy metals [104,105]. Moreover, GSH and PC enhance the majority of ABC transporters in the transfer of Cd [106,107]. 

Since GSH aids Cd exporters and importers in Cd transportation, the observed interface of Cd accumulation could be related to the different forms of its carriers. In addition to the reduced Cd stress, Cd^2+^ was separated by the Cd exchangers inside the vacuole, which may have been the primary Cd tolerance mechanism. With GSH, increased Cd^2+^ was delivered to the vacuole through the cytosol and, thus, more Cd^2+^ entered further into the cell cytoplasm along the outside of the rhizome via the ion contour, which is similar with the findings rice [108].

This tolerance framework mitigates Cd toxicity, mostly in the cytosol. Nevertheless, the most Cd also generates throughout the plant. When Chinese cabbage was subjected to moderate and elevated conditions, Cd expulsion played the most important role in Cd toxicity resistance; with GSH, more Cd^2+^ was transferred, even outside the cell, by Cd transporters in the root cellular membranes, as was noted in Arabidopsis [109]. Furthermore, GSH has a significant effect on iron nutrition, as well as the true function of most “Cd transporters”, which is to transfer some iron ions. As a result, Cd^2+^ is the iron ions’ contender. GSH aids Cd^2+^ absorption, and many more iron particles must be exempt outside of the Chinese cabbage, leaving more nutritional iron ions in the soil. Thus, GSH played various roles in the Cd detoxification of Chinese cabbage [110].

### 4.6. Drought Stress

Drought frequently has limiting impacts on plants’ growth and developmental stages and induces growth inhibition and low total crop production [111]. Stomatal conductance, CO_2_ penetration, membrane electron transfer rate, photosynthesis, and carboxylation efficiency are hampered by drought, and this results in the production of ROS, which triggers oxidative damage. Exogenous supply of GSH attenuated drought stress in plants and was associated with enhanced photosynthesis as well as chlorophyll content and the activity of antioxidant enzymes [112]. In mung beans, GSH treatment reduced drought-induced oxidative stress by boosting glyoxalase activity as well as the antioxidant defense system (Table 1).

Plant hormones, recognized as essential in the system of crop development both under ordinary and stress situations, interface with GSH and its lytic enzymes. Plant hormones such as methyl jasmonate, SA, auxin, ETH, ABA, brassinosteroids, and cytokinin can increase the expression of GST in crops [113].

GST (glutathione S-transferase) overexpression in *Arabidopsis thaliana* has a signaling function and controls plant growth by sustaining GSH pools. In comparison to the wild type, the mutated species atgstu17 produced more GSH and ABA. Additionally, adding exogenous GSH into wild-type plants increased their ABA concentration, produced a phenotype comparable to that of the variant, and improved their ability to withstand drought. The phytohormone ABA, which regulates stomatal aperture, inhibits transpiration rate, and regulates germination, was increased by GSH treatment [114].

In *Ctenanthe setosa* (Marantaceae), drought stress led to leaf rolling; this leaf rolling is regarded as an adaptability associated with increased GSH content as well as reduced GSSG concentration. The AsA–GSH cycle proteins and GSH levels were linked to the elimination of ROS. The stabilization of leaf moisture content was likewise linked to a higher GSH content, suggesting that it may play a role in preventing leaf rolling [115]. 

As increased endogenous GSH levels promote cell proliferation in the root meristem area, which is a key structural response to drought, GSH is thought to have growth-regulating properties. Enhanced endogenous GSH concentrations have been shown to increase resistance to the effects of drought stress and to mitigate the harm caused by ROS in numerous investigations. Substantial oxidative stress in *B. napus* was caused by drought, as seen by a sharp increase in H_2_O_2_ and lipid peroxidation rates. The seedlings’ GSSG activity considerably increased during drought stress. Since it decreased oxidative stress and enhanced drought stress resistance by raising endogenous GSH levels, exogenously administered selenium had a positive impact. Additionally, the GSH/GSSG ratio and AsA–GSH cycle enzyme activity were also raised [116,117].

**Table 1:** Glutathione effect against abiotic stress in various crops using exogenous GSH application.

Abiotic Stress	Crop/Plant	Glutathione Application	Method	Result	Reference
Heat stress	Wheat	-	Endogenously enhanced (GR)	Enhanced tolerance to heat stress	[118]
	Cucumber Mustard	N/A N/A	External application External application	Enhanced heat resistance, plant growth, chlorophyll content, and photosynthetic rate Improved osmoprotectants, ROS detoxification	[67] [119]
Salinity stress	Pepper	0.4 and 0.8 mM	Foliar spray	Enhanced growth and development by improving antioxidant defense system	[120]
	Soybean	1 mM	Foliar application	Increased photosynthesis and antioxidant enzyme activity	[121]
Cold stress	Rice	0.5 mM	GSH application via spraying	Improved growth and development by enhancing GSH level	[122]
	Pepper	0.5 mM	Spraying	Increased endogenous GSH content	[77]
Drought stress	Mung bean	N/A	Exogenous application	Increased antioxidant system; decreased oxidative damage	[123]
	Arabidopsis	N/A	Spraying	Improved drought tolerance	[115]
Heavy metal stress	Maize	30 µM	Foliar application	Enhanced Secondary metabolite and tolerance to cadmium metal stress,	[124]
	Wheat	20 µM	Foliar spray	tolerance to heavy metal stress; increased endogenous GSH content	[102]

GR—Glutathione reductase.

## 5. GSH Interaction with Phytohormones

Phytohormones such as ABA, SA, ET, and JA—which are fundamental for plant development regulation—are, by and large, available in near to the ground fixations but are also important for the improvement, generation, and endurance of plants, in addition to their roles as signaling molecules. Adjustment in phytohormonal concentrations under environmental stresses prompts an intricate crosstalk that assists the plants in undertaking the necessary versatile responses [125,126].

Past research studies have displayed hormonal immune signals which cause a broad transcriptional reorganization, resulting in a well-organized protective response [127]. In plants, for the improvement of environmental stresses, our past studies on proteo-genomics additionally settled the GSH interchange in the company of ABA, along with SA [128,129]. In any case, at the transcriptional level, the outcome of phytohormones lying on GSH is still under investigation. At the transcriptional level, the current study focuses on illustrating the relationship between GSH and numerous phytohormones in samples of Arabidopsis with altered GSH content, such as the GSH-exhausted mutant, alongside the wild-type Col-0, the enhanced pad2-1 (treated with JA, SA, ABA, and ET), and the transgenic AtECS1 line with improved GSH content. This assisted us in acquiring a top-to-bottom comprehension of the connections of GSH with natural phytohormones, namely JA, SA, ABA, ET, and so forth, to alleviate plants resistance.

Salicylic acid (SA), which is a molecule of endogenous immune-signaling nature, plays an effective role in actuating infection-tolerant responses in plants [130]. As a vital controller of SA-actuated systemic acquired resistance (SAR), an increase in SA concentration influences the decrease in disulfide bonds situated on NPR1 (nuclear pathogen-related protein). From the cytosol, NPR1 moves to the nucleus by endless supply of SAR [131]. 

Exogenous SA might, therefore, be used to mimic the state of plants after they have been exposed to biotic and abiotic stimuli that have resulted in increased endogenous SA levels. After SA treatment, the existence of enhanced GSH levels in AtECS1 may accordingly be worthwhile, as articulation of SA-interceded PR gene expression is caused by it, in contrast with the mutant plants and wild-type plants. Discernibly, the acceptance of the genes was not as substantial in pad2-1 in contrast with wild-type plants. In this way, GSH is found to be fundamental for plants in order to confer the resistance towards stress; furthermore, the upgraded GSH via SA signaling may provide protection against stress, as proven in transgenic tobacco from a past examination, which triggers the overexpressing γ-ECS [132].

Essentially, in the contaminated region, *Magnaporthe oryzae*-tainted rice leaves additionally instigated the creation of phytoalexin as well as JA-Ile [133]. Additionally, exogenous JA application affects a few other plants’ stress responses [134]. However, in plants, in this manner, the stress-resistant property initiated through jasmonates may be improved in the presence of GSH (as is proved from articulations of ACO1 in AtECS1, AOS, and PR4 that may be undermined in pad2-1—which, in comparison with Col-0 and AtECS1, has lower expressions of these genes). In the case of treated plants, the expression of PR1 as well as NPR1 is similar in pad2-1, Col-0, and AtECS1; moreover, it reinforces the adversarial activities of signaling in JA as well as in SA [135].

Increased endogenous ET creates increments during specific phases of development, and improvement, for example, senescence, seed germination, abscission, ripening of fruits and, furthermore, reduces the impact of stresses such as drought, flooding, chilling injury, chemical inducers, microbe contamination, and physical injury [136,137]. Plants treated with ET have also been shown to be less susceptible to *Botrytis cinerea* [138]. During stress, increased ET concentration may initiate the safeguard, signaling the alleviation of stress in plants [139]. Consequently, transgenic AtECS1 with improved GSH levels may acquire a benefit to counter the responses of stress, which may be interceded by ET pathways, as was detailed before in transgenic tobacco [18,132].

## 6. Crosstalk between GSH and Jasmonate

Cross-resilience is the imposed resistance to extra abiotic and biotic challenges after focusing on a specific oxidative stress, and it is a limitless protective mechanism in higher plants. *Pseudomonas syringae* becomes impervious when Arabidopsis is treated to ozone in advance [140]. The pre-exposure of tobacco to ozone and ultra-violet light initiated protection from tobacco mosaic infection [141]. Various reports showed H_2_O_2_-prompted stress resistance [142,143]. Tobacco exhibited JA-actuated cross-resilience as well as ozone stress injuries [144]. The cross-resistance induced by JA is clarified by after-effects of upregulated JA-initiated metabolic genes of GSH. JA unequivocally invigorated gene expressions for GSH reuse and synthesis, conceivably prompting upgraded GSH synthesis, giving assurance towards ozone as well as towards oxidative stress. 

Externally applied jasmonates significantly increased transcript modifications, although, under these conditions, no increase in GSH level was seen. GSH homeostasis remains a promising avenue of research and is expected to cover various applications. When there is zero GSH interest, if at that time JA improves the limit with respect to GSH synthesis, when a life form is subjected to oxidative stress, GSH synthesis is predicted to be quicker and more sensitive. [145] recommended that different regulatory “hardware” be engaged for signals of oxidative stress for the purpose of its detection and preparation. The signals originating from various oxidative stress events are frequency-modulated towards the nucleus, where gene expression is initiated, which determines cascade events. In both biotic and abiotic stressors, ROS, GSH, and redox status were hypothesized as focal segments of signal transmission [143]. In light of the fact that the results do not support reactive oxygen as measured by H_2_O_2_ decrease or perhaps the redox potential as measured by the GSH/GSSG ratio as signals framework, under oxidative stress, it is unclear if these putative signal molecules affect the outflow of GSH metabolic genes [143].

The inhibitor proteins jasmonate ZIM domain protein (JAZ) and MYC2 are also highlighted in the review as essential components in crosstalk. JA regulates plant development, abiotic stress tolerance, and defensive resistance against hemibiotrophic diseases such as *Magnaporthe oryzae* and *Pseudomonas syringae* through interacting with other hormone signaling pathways including those of ET, auxin, SA, GA, ABA, and BRs to regulate plant phenology and tolerance to abiotic stress and diseases such as hemibiotrophic pathogens (e.g., *Magnaporthe oryzae* and *Pseudomonas syringae*). In the phytohormone signaling network, JA may function as a key signal [146].

## 7. GSH interaction with Salicylic Acid

Plants experience a wide variety of stresses for the duration of their life in today’s threatening climate. To conquer these unfavorable circumstances, plants employ an assortment signaling molecules. The currently identified molecules are ROS, ABA, SA, ET, and JA, which are used by plants to combat different natural stress circumstances. Over the last two decades, GSH has acquired significance and has become an important molecule for plant scientists, particularly in the field of natural ecological stress organization. Albeit the role of GSH in safeguarding plants has been known for quite some time, a shortage of data still exists; furthermore, in regard to how the system of GSH partakes in this perplexing situation, our investigation shows an interchange in GSH by means of different signal-producing particles such as ABA, SA, and ET, utilizing genetic engineering to deal with, create, and build up transgenic tobacco, which is associated with overexpressing γ-ECS, resistance potential due to biotic/abiotic stresses, and improved GSH concentrations. Transcriptomic profiling distinguished genes as well as proteins identified with ET. SA was also associated with the potential related to stress resistance [147].

In plants, in responsive mechanisms instigated from different microbes, SA acts as a significant signaling molecule which plays a fundamental job [148]. Ongoing information points to the fact that, for regulating plant reactions towards numerous environmental stresses, SA additionally plays significant roles in response to environmental stresses include chilling, heat, salinity, and drought stresses. Conversely, abiotic resistance mediated through SA remains underappreciated, and investigations on SA-mediated abiotic resilience has primarily been conducted at the physical level. SA may have acted as a scavenger for eliminating ROS created during abiotic stress [149].

Antioxidant enzymatic frameworks (for example, peroxidase (POD), superoxide dismutases (SOD), as well as catalase (CAT)) as well as substances such as GSH and AsA have been linked to protection from ROS induced through stress conditions such as salt stress [150,151]. Earlier studies have shown that, SA strikingly increased AsA along with GSH levels as well as the gene that encodes the AsA–GSH cycle and its enzyme under cold stress conditions in maize, cucumber and rice, as well as in eggplant; researchers concluded that SA-prompted cold resistance has a significant role in the AsA–GSH cycle [152,153]. Various plant types have evolved immune processes to various biotic and abiotic stresses [154]. Conversely, during salinity, as far as anyone is concerned, at a molecular level, no examination has been accounted for on the connection between SA and synthesis of the AsA–GSH cycle in higher plants. To study the relationships between SA-instigated salinity resilience and AsA–GSH production at the molecular level in higher plants, the transcriptional activity (including its target gene which encodes AsA–GSH cycle molecules) in SA-treated wheat plants undergoing salinity stress was evaluated. Utilization of SA in a 0.5 mM concentration in green gram increased the action of photosynthesis under salt stress conditions, and increased antioxidant protein action in addition to GSH concentration [155,156].

## 8. GSH Defense Enzymes in Crops

It has been suggested that the alkylation of the cysteine residue in GSH, proceeded by dissociation and oxidation to create alkenyl cysteine sulphoxide, is the first step in the production of taste precursors [157]. The catalytic activity of several GSH-dependent enzymes, such as GSTs and glyoxalase-I, use the tripeptide GSH as a detoxifying agent for both endogenous and exogenous toxins. In comparison, L-cystine conjugates are quickly metabolized in the vacuole from GSH conjugates, which are created by the catalytic properties of GSTs [158]. Alliinase or other such enzymes then proceed to metabolize cysteine conjugates to thiol and other related compounds in the cytosol following their efflux from the vacuole [159]. All of these events imply that GSTs, glyoxalase-I, and alliinase are interconnected. While analyzing the three enzymes’ activity in soluble vegetable extracts, a relationship between them was determined. The greatest GST activity was observed in onions, and then in Western blotting, a broad band for presumptive GST activity was recognized by anti-CmGSTF1 antiserum. The dissolved extraction, meanwhile, displayed the highest specified level of activity for onion glyoxalase-I, suggesting that the band may have partially been caused by the enzyme.

Detoxifying enzymes such as GSTs and glyoxalase-I serve a purpose for GSH. As a result, onion bulbs should exhibit the highest levels of these enzymes’ activity, which may indicate a connection between the two enzymes. Additionally, alliinase, a separate type of enzyme whose activity is relevant to sulfur compounds formed from GSH, demonstrated the highest degree of activity in onion bulbs [157]. The primary role of GSTs is the detoxification of endogenous and herbicidal toxins; hence, the presence of proteins in vegetables may improve food quality. Food quality may be connected with glyoxalase enzymes which detox harmful methylglyoxal. Although there are studies on the features and activity of individual enzymes in a single plant, it is difficult to compare the activities of GST, glyoxalase-I, and alliinase in one plant or a group of plants. Concentrations of the three-enzyme activity in various vegetable crops recovered under the same conditions [157,160].

The earlier studies show that some transgenic crops regulate GSH levels by enhancing the enzymes activities and ultimately enhance the tolerance against biotic and abiotic stresses (Table 2).

## 9. GSH Role in Root Architecture and Root-Derived Changes in Shoots

In addition to modulating physiological and metabolic functioning in plant’s foliar parts, GSH takes part in modifying root traits and/or root-to-shoot signaling, thereby affecting shoot attributes. GSH’s involvement in root development is prevalent but its functions are poorly understood. Suppression in root growth, structural changes (length and cell division) in root apical meristem, and abnormalities in lateral root formation were observed when plants were treated with the GSH biosynthesis inhibitor buthionine sulfoximine (BSO) and Arabidopsis mutants lacking GSH biosynthesis (cad2, rax1, and rml1) [168]. The molecular mechanisms of GSH action were studied, and it was discovered that GSH deficiency affected the total ubiquitination of proteins and inhibited the transcriptional activation of early auxin-responsive genes as well as the auxin-related, ubiquitination-dependent degradation of Aux/IAA proteins. The ROS control of GSH, GSH’s active participation in cell proliferation, and GSH’s interactions with auxin suggest that GSH may modulate root development under stress conditions [169]. A study demonstrated a novel role for GSH in the regulation of root architecture connected to auxin and strigolactone signaling. Furthermore, the findings show that BSO-dependent suppression of GSH synthesis does not result in the same root architecture phenotype as was seen in mutants that were GSH synthesis-defective. BSO had little effect on lateral root density, while all GSH synthase mutants greatly reduced it. The contrasts between these results and those in cells where GSH synthesis is hindered by BSO may be explained by variations in the intracellular distribution of GSH in the mutants [170].

The root-derived restricted movement of a toxic metal, cadmium (Cd), to shoots with GSH treatment was demonstrated by researchers while studying the effect of exogenously applied GSH (to roots and shoots) on the distribution and behaviour of cadmium (Cd) in roots of *Brassica rapa* [171]. The Cd moving from roots to shoots was preferentially prevented by GSH administration to the root medium, but was not affected when applied to the shoots, rather, the Cd level in shoots was slightly increased by foliar application especially under long-term Cd exposure conditions. Moreover, no change in root Cd concentration occurred by any means of GSH application to *Brassica* plants. The reduced root-to-shoot Cd transfer in the presence of GSH in the root zone was attributed to reduced Cd concentration in symplast sap xylem vessels, as well as to the activation of Cd efflux from root cells under GSH treatment [171]. Furthermore, it is evident that GSH application affects root contents of phytochelatins (PCs), which play crucial roles in heavy metal sequestration in vacuoles and their movement from root to shoot [107,172,173]. Hence, from this, it is clear that GSH not only affects the root traits but also helps modify the root-derived shoot traits under stressful conditions when applied appropriately.

## 10. Conclusions

The impact of stress can prompt inadequacy in plant growth and development, crop yield, and continuous harm if the stress prolongs beyond plant resistance limits. Most plants gain resilience to mild stress when they are exposed to the stress. During abiotic stresses, actuation of transcriptional factors and activation of stress-responsive genes happen through signaling transduction and, hence, these components control the harm suffered due to stresses and provide resistance in plants. GSH assumes a critical role in abiotic stress resistance by improving plants’ resilience to various stresses. It assists with combating free radicals, for example, it detoxifies ROS in plants, enhancing antioxidative defense mechanism and modifying root architecture and root-derived effects in overcoming stress impacts. Nonetheless, results concerning general stress-related responses are still very irregular. In future, exploration studies should focus on the crosstalk among GSH and other signaling phytohormones during abiotic stresses as very little work has been conducted on this aspect. GSH could lower H_2_O_2_ and ROS production during oxidative stress. It is expected that the study of stress tolerance in crops will develop further in the future to achieve great agricultural yield and ensure food and nutritional securities under rapidly changing climatic conditions.

## Figures and Tables

**Table 2:** Glutathione responses for cold tolerance with transgenes.

Target Species	Common Name	Gene	Glutathione Content	Physiological Effect after Transgenic	Experimental Conditions in Pot or Soil Bed	References
*Solanum lycopersicum*	Tomato	*DREB1*	Increase	Antioxidant machinery effectively activated	Greenhouse experiment	[161]
*Nicotiana tobacum*	Tobacco	*DHAR:GR*	Increase	Enhanced tolerance against cold stress	Pots for rooting then moved to soil for seed collection	[162]
*Arabidopsis thaliana*	Arabidopsis/thale cress	*GSH1*	Increase (GSSG/GSH)	Vernalization	Pots experiment	[163]
*Brassica napus*	Rapeseed	*GSTF11*	Decrease (GSH:GSSG)	Increased resistance to powdery mildew	Experimental plot and laboratory	[164]
*Chrysanthemum*	Crysanths	*DgGPX1*	Increase Glutathione peroxidase	Reduced ROS generation, Cold tolerance enhanced.	Pots experiment	[165]
*Salvia miltiorrhiza*	Red sage/Danshen	*RcGPX5*	GSH and total glutathione concentration higher	Increased tolerance to oxidative stress	Controlled conditions, and then introduced to field	[166]
*Arabidopsis thaliana*	Arabidopsis	*OsGSTL2*	Overexpression of GSTs	Tolerance to heavy metal and abiotic stress	Under lab conditions	[167]
*Populus trichocarpa*	Poplar	*PtGSTF1*	Overexpression of Glutathione S-transferase	Improved salt resistance, ROS scavenger	Pot experiments in greenhouse	[168]

## Data Availability

Not applicable.
